# Supplementary material for: Viral Co-Infection in Bats: A Systematic Review
Source: Viruses. 2023 Aug 31;15(9):1860. doi: 10.3390/v15091860 (PMC10535902; doi:10.3390/v15091860)
Supplement: Supplementary file 1 [file viruses-15-01860-s001.zip › Supp_file_S1.pdf]

Supplementary File 1
Search strategies – 2/12/2021

Embase via Elsevier

#4	#1 AND #2 AND #3	2,283
#3	study:ti,ab,kw OR studies:ti,ab,kw OR survey:ti,ab,kw OR surveillance:ti,ab,kw OR investigation:ti,ab,kw OR detection:ti,ab,kw OR coexist*:ti,ab,kw OR 'co-exist*':ti,ab,kw OR coinfect*:ti,ab,kw OR 'co-infect*':ti,ab,kw OR 'co-circulat*':ti,ab,kw OR cocirculat*:ti,ab,kw OR concurrent*:ti,ab,kw OR concomitant*:ti,ab,kw OR polyinfect*:ti,ab,kw OR polyparasitism:ti,ab,kw OR ((infection NEAR/4 (multiple OR mixed OR simultaneous OR combined)):ti,ab,kw)	15,865,332
#2	viral*:ti,ab,kw OR virus*:ti,ab,kw OR virulen*:ti,ab,kw OR virology:ti,ab,kw	1,271,519
#1	chiroptera*:ti,ab,kw OR bat:ti,ab,kw OR bats:ti,ab,kw OR 'flying fox*':ti,ab,kw OR 'flying-fox*':ti,ab,kw	25,251

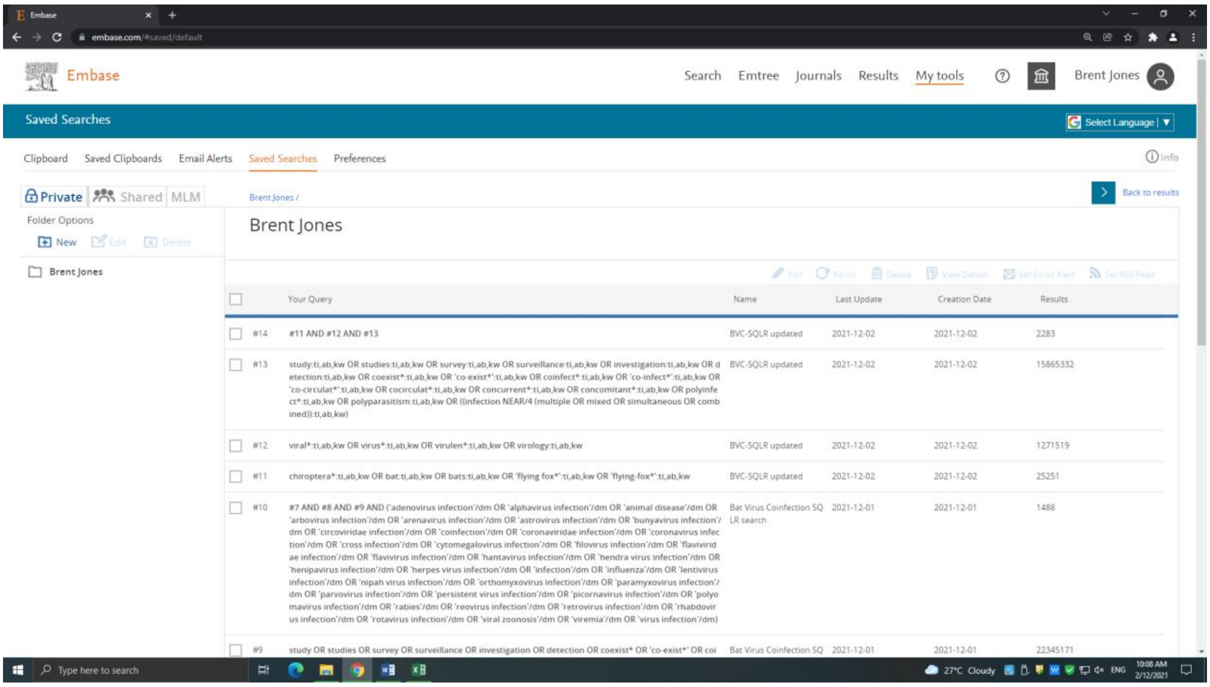

Scopus

(TITLE-ABS-KEY (chiroptera* OR bat OR bats OR "flying fox*" OR flying-fox*)) AND (TITLE-ABS-KEY (viral* OR virus* OR virulen* OR virology)) AND (TITLE-ABS-KEY ((study OR studies OR survey OR surveys OR surveillance OR investigation OR detection OR coexist* OR co-exist* OR coinfect* OR co-infect* OR cocirculat* OR co-circulat* OR concurrent* OR concomitant* OR polyinfect* OR polyparasitism) OR (infection W/4 multiple OR mixed OR simultaneous OR

combined))) AND (LIMIT-TO (EXACTKEYWORD , "Bat") OR LIMIT-TO (EXACTKEYWORD , "Chiroptera")) AND (LIMIT-TO (EXACTKEYWORD , "Virology") OR LIMIT-TO (EXACTKEYWORD , "Virus Transmission") OR LIMIT-TO (EXACTKEYWORD , "Virus Replication") OR LIMIT-TO (EXACTKEYWORD , "Virus Infection") OR LIMIT-TO (EXACTKEYWORD , "Virus Identification"))

1,534 results

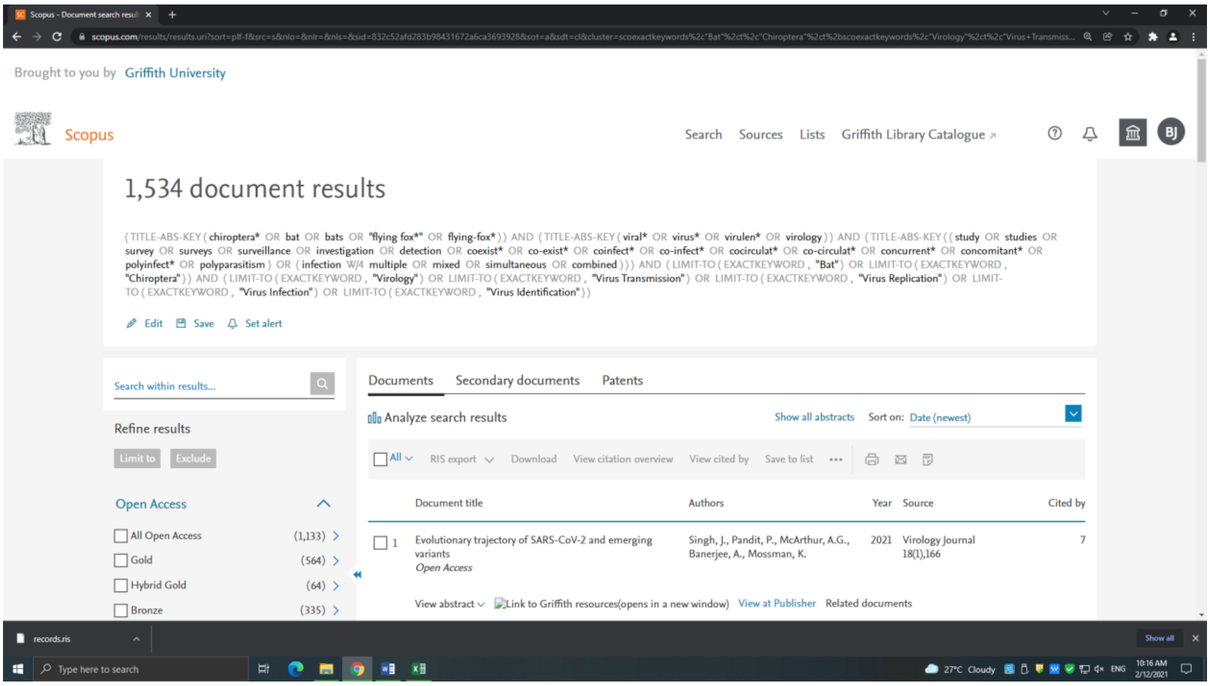

Web of Science

#5	#1 AND #2 AND #3 and Virology (Web of Science Categories)	761
#4	#1 AND #2 AND #3	2,844
#3	TS=((study OR studies OR survey OR surveys OR surveillance OR investigation OR detection OR coexist* OR co-exist* OR coinfect* OR co-infect* OR cocirculat* OR co-circulat* OR concurrent* OR concomitant* OR polyinfect* OR polyparasitism) OR ((multiple OR mixed OR simultaneous OR combined) AND infection))	23,409,584
#2	TS=(viral* OR virus* OR virulen* OR virology)	1,294,438
#1	TS=(chiroptera* OR bat OR bats OR "flying fox*" OR flying-fox*)	44,478

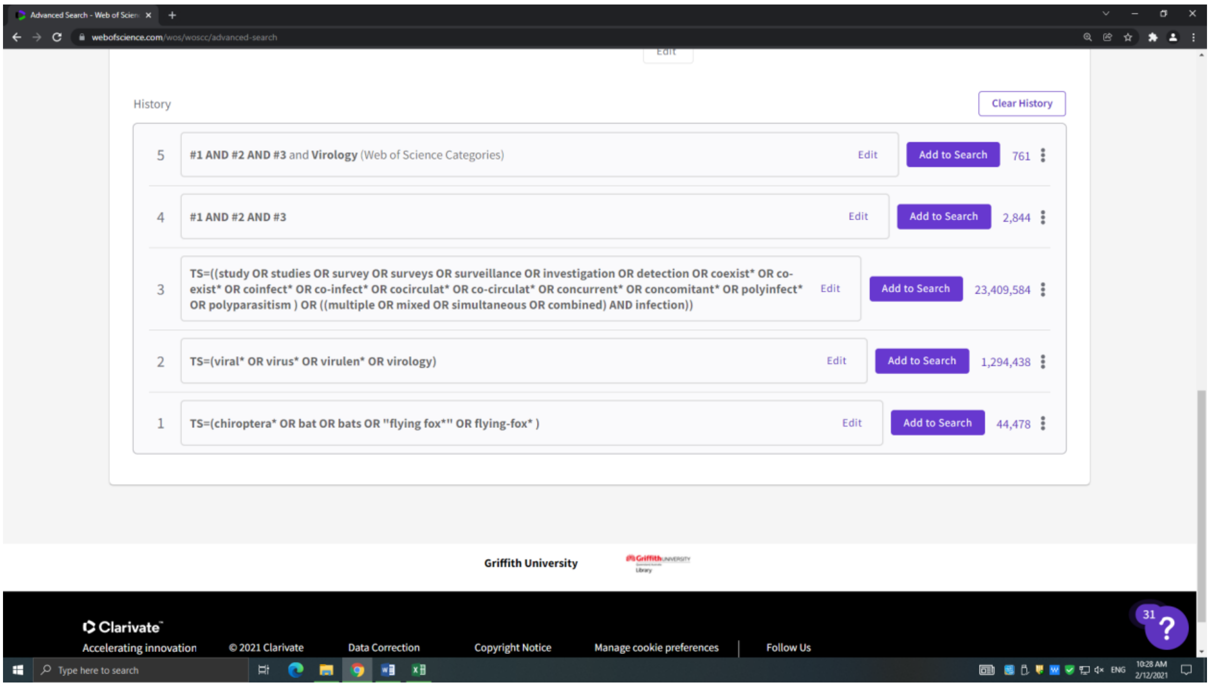

PubMed

#4	#1 AND #2 AND #3	2,184
#3	(study[Title/Abstract] OR studies[Title/Abstract] OR survey[Title/Abstract] OR surveys[Title/Abstract] OR surveillance[Title/Abstract] OR investigation[Title/Abstract] OR detection[Title/Abstract] OR coexist*[Title/Abstract] OR co-exist*[Title/Abstract] OR coinfect*[Title/Abstract] OR co-infect*[Title/Abstract] OR cocirculat*[Title/Abstract] OR co-circulat*[Title/Abstract] OR concurrent*[Title/Abstract] OR concomitant*[Title/Abstract] OR polyinfect*[Title/Abstract] OR polyparasitism[Title/Abstract]) OR ((multiple[Title/Abstract] OR mixed[Title/Abstract] OR simultaneous[Title/Abstract] OR combined[Title/Abstract])AND infection[Title/Abstract])	12,359,109
#2	viral*[Title/Abstract] OR virus*[Title/Abstract] OR virulen*[Title/Abstract] OR virology[Title/Abstract]	1,072,053
#1	chiroptera*[Title/Abstract] OR bat[Title/Abstract] OR bats[Title/Abstract] OR "flying fox*"[Title/Abstract] OR flying-fox*[Title/Abstract]	21,213

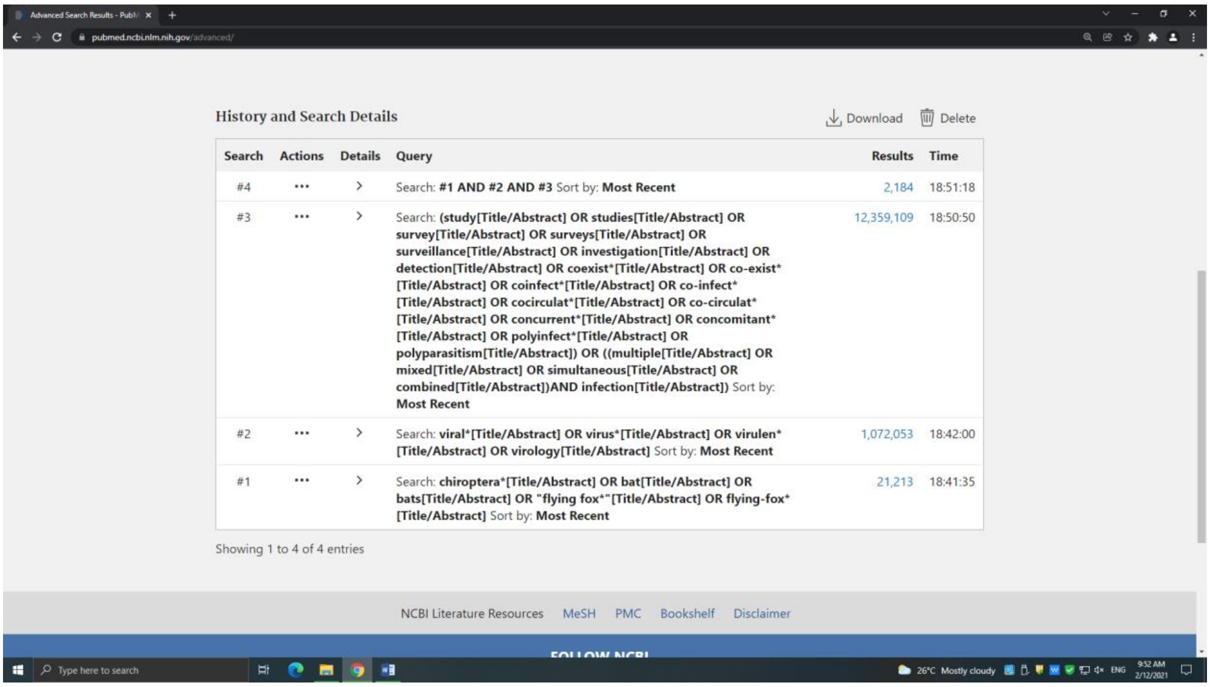

GreenFILE via EBSCOhost

TX (chiroptera* OR bat OR bats OR "flying fox*" OR flying-fox* OR DE "BAT conservation") AND TX (viral* OR virus* OR virulen* OR virology OR DE "VETERINARY virology" OR DE "VIRUSES" OR DE "VIRUS diseases") AND TX ((study OR studies OR survey OR surveillance OR investigation OR detection OR coexist* OR co-exist* coinfect* OR co-infect* OR co-circulat* OR cocirculat* OR concurrent* OR concomitant* OR polyinfect* OR polyparasitism) OR (infection N4 multiple OR mixed OR simultaneous OR combined) OR DE "INFECTION" OR DE "INFECTIOUS disease transmission")

78 results

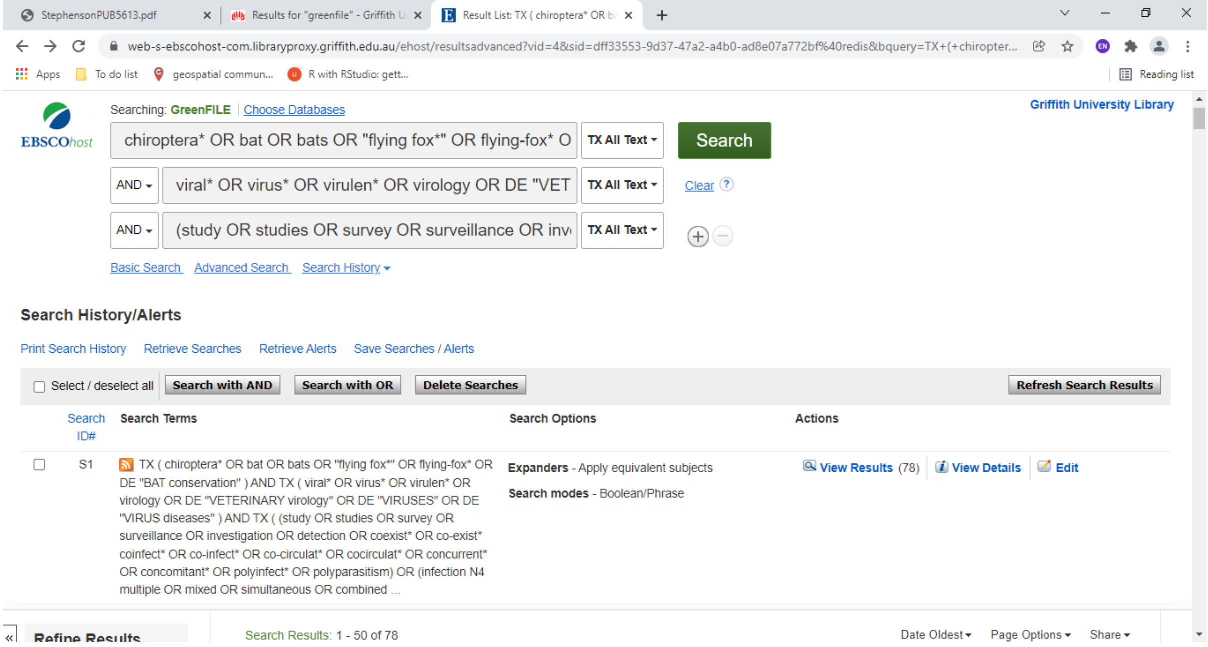

ProQuest (Databases: Biological Science, Environmental Science, Public Health)

noft(chiroptera* OR bat OR bats OR "flying fox*" OR flying-fox*) AND noft(viral* OR virus* OR virulen* OR virology) AND noft(((study OR studies OR survey OR surveys OR surveillance OR investigation OR detection OR coexist* OR co-exist* OR coinfect* OR co-infect* OR cocirculat* OR co-circulat* OR concurrent* OR concomitant* OR polyinfect* OR polyparasitism) OR (infection NEAR/4 multiple OR mixed OR simultaneous OR combined))) AND subt.exact("viruses" OR "bats" OR "chiroptera")

1,113 results

*subject filter for viruses or bats or chiroptera

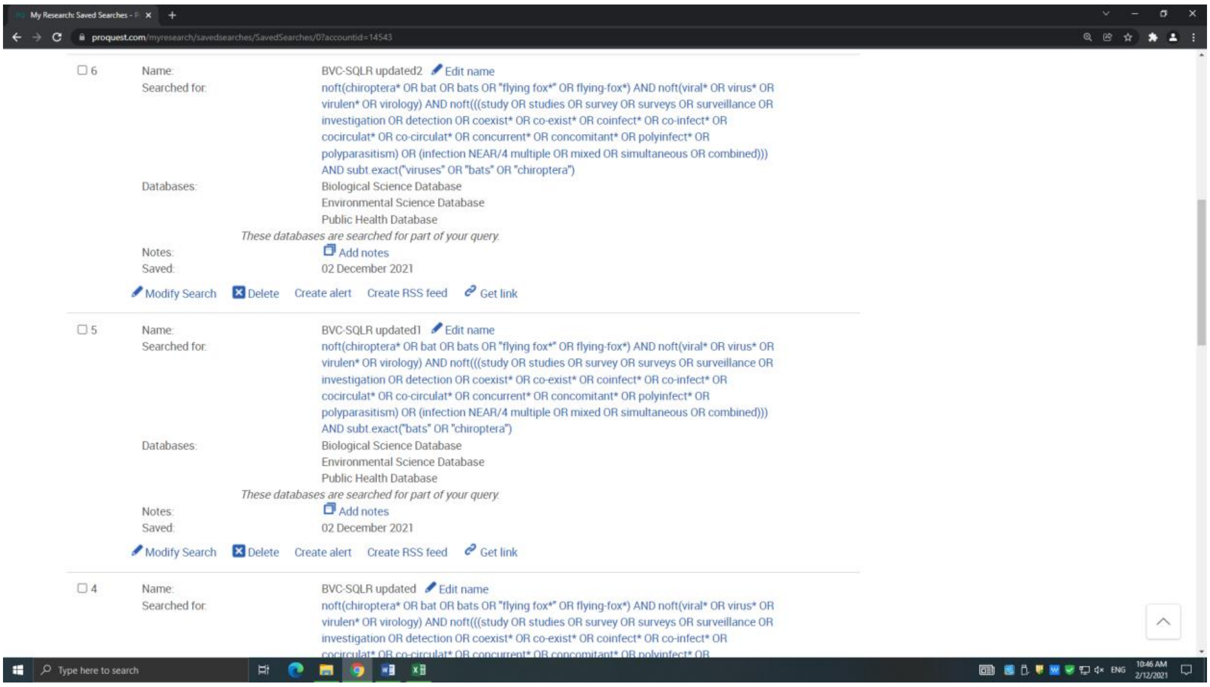

Figures S1-S4

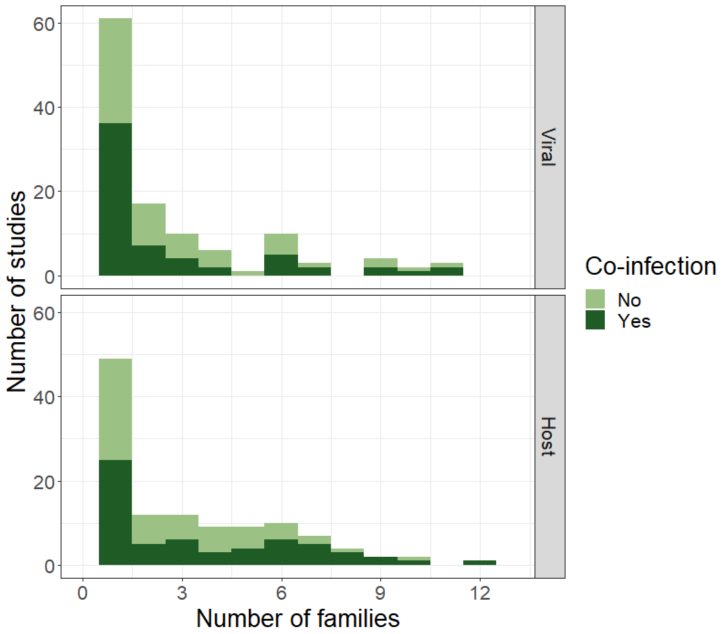

Figure S1: Bar chart displaying the number of viral (top chart), and host (bottom chart) families screened per study, grouped by whether or not they detected co-infection.

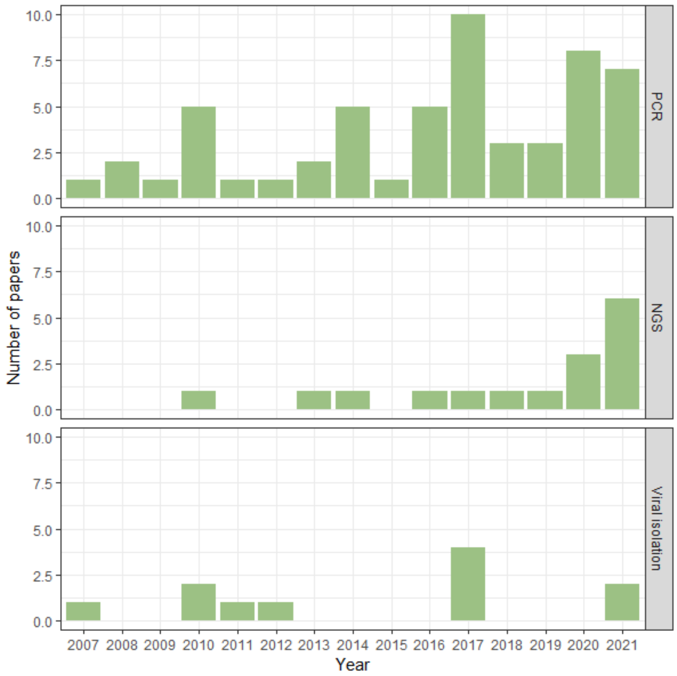

Figure S2: Viral detection method usage over time in publications detecting co-infection in the database.

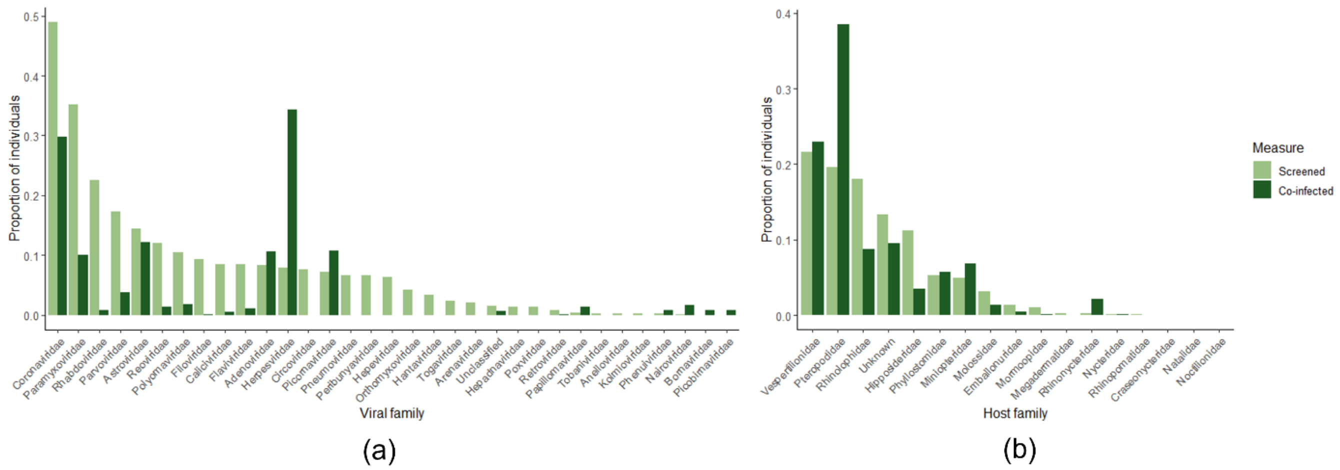

Figure S3: Proportion of individuals in the database that were screened for and detected with co-infection from each viral (a) and host family (b). The proportion of individuals screened is from the total number of individuals in the database while the proportion co-infected is from the total number of co-infected individuals in the database.

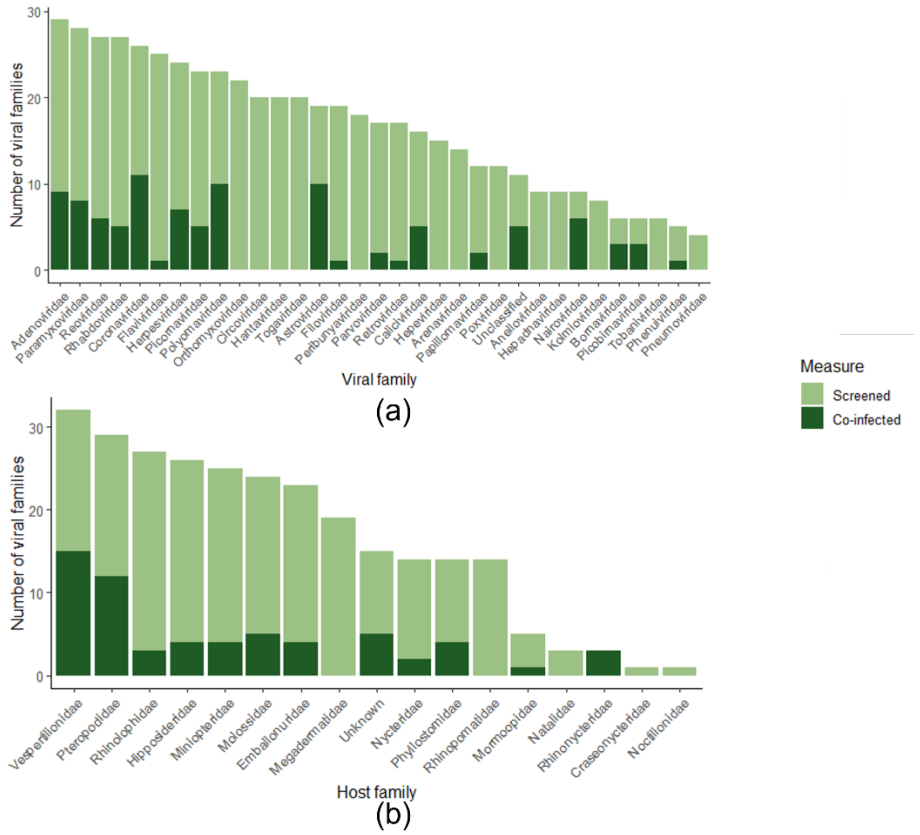

Figure S4: Bar chart displaying the number of viral families each individual viral (a) and host (b) family was screened against, grouped by whether co-infection was detected or not.
